# Immune-Mediated Repair: A Matter of Plasticity

**DOI:** 10.3389/fimmu.2017.00454

**Published:** 2017-04-24

**Authors:** Paôline Laurent, Valérie Jolivel, Pauline Manicki, Lynn Chiu, Cécile Contin-Bordes, Marie-Elise Truchetet, Thomas Pradeu

**Affiliations:** ^1^ImmunoConcept, UMR5164, Immunology, CNRS, University of Bordeaux, Bordeaux, France; ^2^Rheumatology, CHU Bordeaux Hospital, Bordeaux, France; ^3^Immunology, CHU Bordeaux Hospital, Bordeaux, France

**Keywords:** repair, plasticity, robustness, fibrosis, macrophages, neutrophils, innate lymphoid cells, transdifferentiation

## Abstract

Though the immune system is generally defined as a system of defense, it is increasingly recognized that the immune system also plays a crucial role in tissue repair and its potential dysregulations. In this review, we explore how distinct immune cell types are involved in tissue repair and how they interact in a process that is tightly regulated both spatially and temporally. We insist on the concept of immune cell plasticity which, in recent years, has proved fundamental for the success/understanding of the repair process. Overall, the perspective presented here suggests that the immune system plays a central role in the physiological robustness of the organism, and that cell plasticity contributes to the realization of this robustness.

“The medical and naturalist philosophers have been vividly struck by this tendency of the organized individual to reestablish its form, to repair its mutilations, to heal its wounds, and in this way to prove its unity, its morphological individuality (*1*).”

## Introduction

All organisms possess the crucial property of *robustness*, which is a system’s capability to maintain its functions and performances despite perturbations (2–4). Robustness includes the capacities for each organism to build, defend, and repair itself (1, 5). The immune system, by constantly surveying the body and responding to strong anomalies, plays a key role in robustness (6, 7). In this review, we analyze how the immune system regulates tissue repair and show that one major way by which the immune system contributes to robustness is through immune cell plasticity. Notably, innate immune cells are particularly important in tissue repair, which suggests that the role of immunity in maintaining repair-oriented robustness has been conserved through evolution.

Tissue repair can be defined as the process that restores (at least partially) tissue organization after an insult (8, 9). The insult consists in damages by physical or chemical aggression, infectious agents, or “internal” stresses (10, 11). Tissue repair may be divided into three partly overlapping stages (8, 12). The first stage, *inflammation* (0–48 h after injury), immediately follows tissue damages and triggers the activation of the components of the coagulation cascade, the immune system, and inflammatory pathways. The second stage, *new tissue formation* (2–10 days after injury), is characterized by the proliferation and migration of many cell types and angiogenesis (i.e., formation of new blood vessels), and the progressive restoration of tissue function. Finally, during the third stage, *remodeling* (starts 2–3 weeks after injury and can last several months), many activated cells (e.g., endothelial cells, macrophages, and myofibroblasts) die by apoptosis or leave the site of injury, and tissues are re-epithelized.

The perturbation of one or several of these three stages leads to dysregulated repair and can have important pathological consequences (13). Several different disorders can be described as the result of abnormal repair. Fibrosis, for instance, is often seen as the consequence of “over-repair” or “over-healing” (8, 13), with excessive accumulation of extracellular matrix (ECM) and failure to restore tissue function and architecture (14), as observed in idiopathic pulmonary fibrosis, heart failure, and several autoimmune diseases such as systemic sclerosis (15). In contrast, ulcers can be viewed as the consequence of “under-healing” (16). Cancerous tumors can also be seen as products of an abnormal repair process, or “wounds that do not heal” (17, 18).

**Figure 1:**
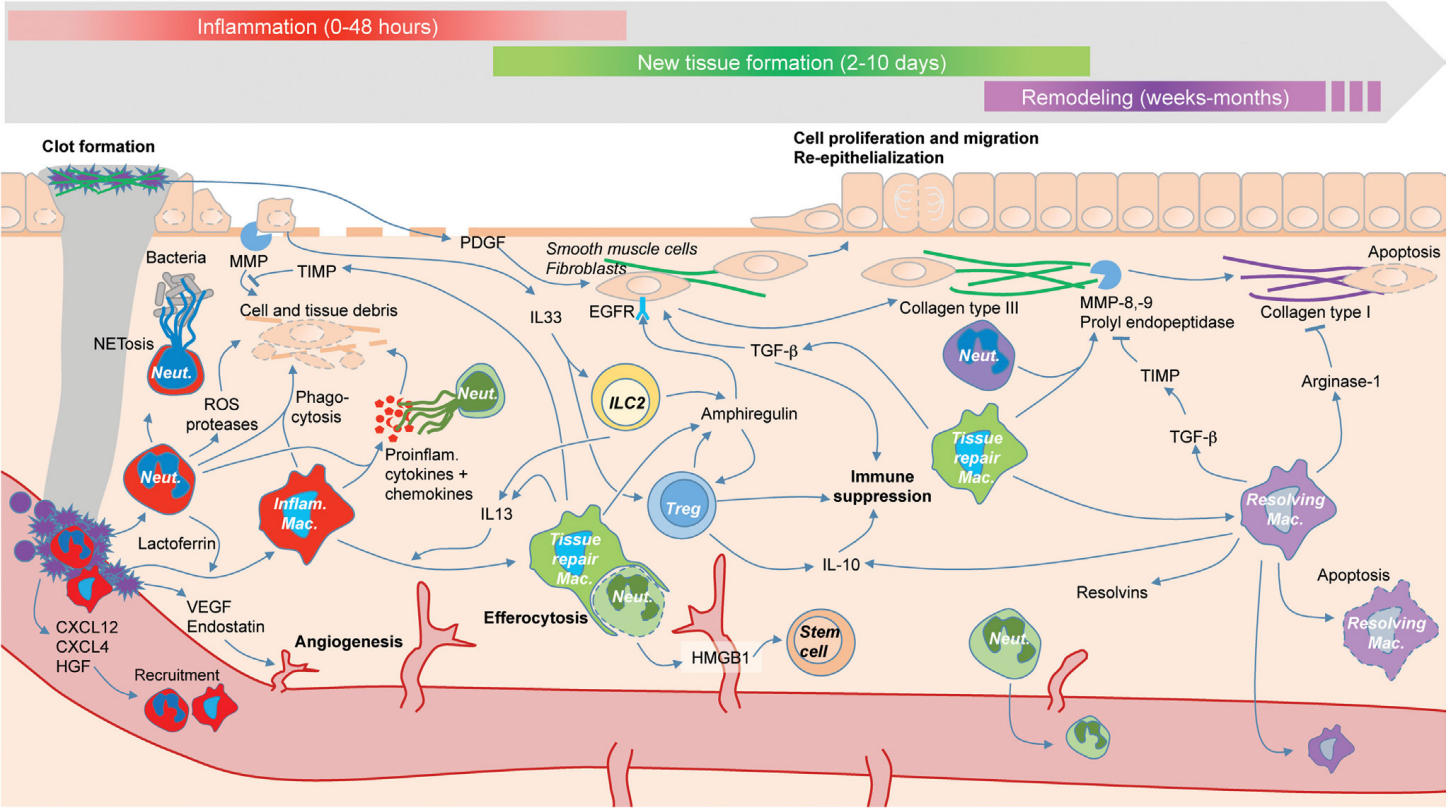
**The roles of immune cells (colored) during the three phases of tissue repair: inflammation (red), new tissue formation (green), and remodeling (purple)**. Tissue repair is driven by interactions between immune cells and other cell types such as fibroblasts (not colored), as well the temporary plasticity of immune cells, e.g., macrophage switch from an inflammatory phenotype (red) to a tissue repair phenotype (green) to a resolving phenotype (purple).

Though the immune system is generally defined as a system of defense, selected through evolution for its capacity to insure host protection (19–23), it is now clear that the immune system plays also an essential role in tissue repair (9, 24–26). The involvement of the immune system in repair had long been suspected (27–30), but it is only recently that a precise cellular and molecular characterization of this process has been possible. In this review, we describe the impact of the immune system on repair and dysregulated repair and emphasize the key role played by immune cell plasticity in repair (Figure 1) (31). The word “plasticity” is used with different and often confusing meanings. Here, we understand cell plasticity in two different and important senses. The first sense is *intra-lineage cell plasticity*, that is, changes in cell function and phenotype within a given cell lineage—for example, type 1 macrophages (M1s) turning into type 2 macrophages (M2s). This is what is sometimes called “functional plasticity” (32). The second sense is *trans-lineage cell plasticity*, that is, the switch from one lineage to another—e.g., from macrophages to fibroblasts (33). This can also be called plasticity by “transdifferentiation” (34) or by “reprogramming”—a phenomenon now known to occur in some non-immune cells (35). We show here how these two types of cell plasticity are involved in tissue repair—with a particular insistence on the first type, which is now amply demonstrated. Because the most numerous and crucial immune cells involved in tissue repair are macrophages and neutrophils, we start our review with a description of their roles and plastic capacities.

## Plasticity of Neutrophils and Macrophages in Repair

Neutrophils are part of the first line of defense against infection and are massively recruited on damage sites (36). The defense function is mainly mediated by phagocytosis, intracellular degradation, releasing of granules, and formation of neutrophil extracellular traps (NETs) after sensing dangerous stress. However, accumulating data show that neutrophils have a variety of important biological functions far beyond cytotoxicity against pathogens. Their adaptable life span, longer than previously thought (more than 5 days) (37), allows neutrophils to participate actively in the three repair stages (38).

Far from being “one-shot” weapons, long-living neutrophils are remarkably plastic. Indeed, neutrophils can differentially switch phenotypes, display distinct subpopulations under different microenvironments, and produce a large variety of cytokines and chemokines (39). Plasticity of neutrophils has been evoked recently following the controversial debate on their pro- or antitumor role, leading to the conclusion that they can be both, depending on the subsets under consideration. In repair, neutrophils can be pro- or anti-resolution of the inflammatory stage. The resolution of tissue formation depends on neutrophils for their ability to (i) produce several pro-resolving mediators (as lipoxins) (40), (ii) form NETs and aggregated NETs, according to a cell-density dependent sensing mechanism, which dismantles the pro-inflammatory gradient by degrading the inflammatory cytokines and chemokines (41), and (iii) store and release the pro-resolving protein annexin A1 (42–44).

In addition to this intra-lineage plasticity, repair-associated neutrophils are capable of trans-lineage plasticity (i.e., plasticity by transdifferentiation) (45, 46). Challenging the classic view of neutrophils as terminally differentiated leukocytes fully committed to phagocytosis, new evidence shows that neutrophils can acquire phenotypic and functional properties typically associated with professional antigen-presenting cells (APCs) (47, 48)—e.g., MHC II expression and co-stimulatory molecules (49). Such neutrophil trans-lineage plasticity is driven in part by the microenvironment composition. For example, in an inflammatory and pro-type 2 environment of a lesion, neutrophils could transdifferentiate into APCs (46). Transformation of neutrophils into APCs has also been studied in rheumatoid arthritis, where it could drive sustained inflammation, thereby preventing normal repair (50). Trans-lineage plasticity could also link innate and adaptive immunity and constitute a pivotal crossroad polarizing the response toward abnormal repair, e.g., fibrosis.

Recent research has also highlighted the plasticity of monocytes and macrophages. During the early inflammatory phase (stage 1), monocytes are attracted by neutrophils to the wound site, where they represent a predominant source of pro-inflammatory mediators and exert a phagocytic role as M1 (51). Early depletion of macrophages in mice leads to decreased inflammatory responses (52).

Type 1 macrophages drive the early inflammatory responses that lead to tissue destruction, whereas M2s (or “alternatively activated reparative macrophages”) exert a central role in wound healing. Both types are needed for proper wound healing (53). The second stage of the repair process—new tissue formation—is dominated by M2s (26, 54, 55). These cells promote tissue repair by producing pro-reparative cytokines or generating a pro-type 2 microenvironment. They also present a complex heterogeneity (56). Beyond a strict M1/M2 dichotomy, a wide range of macrophage subtypes exists (57–59), and several of them are involved in repair (34). Efficient tissue repair requires inflammatory macrophages, tissue repair macrophages, and resolving macrophages (producers of resolvins, IL10, and TGF-b) (26, 34, 60).

During the remodeling phase, most macrophages undergo apoptosis or leave the wound (8). At this stage, the wound consists mostly of an acellular matrix where the collagen type III is substituted by collagen type I, thanks to the secretion of matrix metalloproteinases, notably by tissue repair macrophages. Even if current evidence is limited, macrophages might participate actively in tissue remodeling by transdifferentiation, notably into endothelial cells (61–64).

All this raises the question of whether the crucial and distinct roles played by macrophages and neutrophils in tissue repair are better explained by cell migration or by cell plasticity (65). Do subtypes of macrophages and neutrophils (M1s and M2s; pro-inflammatory and pro-resolving neutrophils) reach the tissue separately, in successive waves, or are they the product of a plastic switch from one subtype to the other? Further research is needed to answer this question.

## Plasticity of Other Immune Cells in Repair

Though neutrophils and macrophages constitute the most numerous immune cells involved in tissue repair, this process also includes other immune cells—particularly γδ T cells, innate lymphoid cells (ILCs), and regulatory T cells (Tregs).

Resident T cells are present in human epidermis with both αβ and γδ subtypes. There is a lot of evidence for the involvement of γδ T cells in repair processes in mice (66). The situation is far less clear in humans. Nevertheless, some studies showed in culture models the promotion of wound healing by γδ T cells, through the production of the insulin-like growth factor 1 (67). Plasticity of γδ T cells has been only evoked in the context of cancer, where γδ T cells display either pro- or antitumor activity depending on the cytokine environment (68, 69). Further investigations are needed to demonstrate if this functional plasticity of γδ T cells is also involved in tissue repair.

Innate lymphoid cells are a recently discovered family of immune cells that includes three subsets: ILC1, ILC2, and ILC3 (70–72). These cells are found preferentially on epithelial barrier surfaces such as skin, lungs, and gut, where they protect against infection and maintain the integrity of the barriers (73, 74). ILCs are tissue-resident sentinels enriched at mucosal surfaces and have a complex crosstalk with the microenvironment. They are highly involved in tissue repair through their sentinel position and the cytokines they produce (75, 76). ILC2-secreted amphiregulin, a protein shown to orchestrate tissue repair (75), promotes wound healing by acting directly on fibroblasts, leading to ECM deposit. Key transcription factors determine the pathway of differentiation of progenitors toward an ILC1, ILC2, or ILC3 subset. ILC responses to different stimuli allow intra-lineage plasticity between the different subsets (77, 78). This plasticity between different ILC subtypes might allow for rapid innate immune responsiveness in repair (79, 80).

Regulatory T cells exert multiple functions (81) and could play a critical role in tissue repair (82). Recently, several populations of tissue-resident Tregs were identified, including special Tregs in visceral adipose tissue, muscle Tregs, and skin-resident memory Tregs (83, 84). In repair, Tregs could modulate the effector response and contribute to the redundant effect of the previously described cells by creating a dynamic microenvironment. Tissue-resident Tregs are able to rapidly respond to tissue damage, inducing tolerance and repair to prevent loss of tissue function. Tregs share these features with ILC2s and anti-inflammatory macrophages.

Thus, many elements are redundant in tissue repair. This redundancy likely illustrates the critical role played by immune-associated repair in the survival and robustness of the organism.

## Cellular Plasticity in Wound Repair: A Promising Avenue for Future Experimental Research

Immune cells participate in the generation of a peculiar microenvironment, leading to a balance shift between the pro-inflammatory and pro-reparative sides of tissue repair. In cystic fibrosis, for example, abnormal intra-lineage neutrophil plasticity has been implicated in the unbalance of airways microenvironment, leading to chronic inflammation and inability for other cells such as macrophages to switch to resolving stages (85). Hence, manipulation of this process constitutes an innovative therapeutic approach for pathological conditions involving dysregulated repair. Here, we explore different examples of therapeutic modulations of intra-lineage plasticity, for both macrophages and neutrophils.

### Targeting Functional Plasticity during Tissue Injury

In certain conditions, accelerating tissue repair could be decisive, particularly for certain wounds (such as large or life-threatening wounds) and for certain patients (such as elderly or fragile patients). Remarkably, patients treated by immunosuppressive therapy experience a delayed wound healing, which shows that the inflammatory stage is important to induce repair. The complexity of tissue repair is due to the number of involved partners but also to the precise timing and imbrication of the phases. Therefore, isolating new targets, even of great importance, will not be sufficient if the whole balance and timing are not considered.

Numerous mediators involved in the phenotype conversion of macrophages have been described, but so far their therapeutic potential remains uncertain in humans (86). For example, *in vitro* studies showed that GM-CSF could switch inflammatory monocytes to a reparative phenotype, leading to the idea that GM-CSF could exert beneficial effects on intestinal inflammation and wound healing associated with Crohn’s disease (87). Intra-lineage plasticity of macrophages could also be modulated through the VEGF-C/VEGFR3 pathway, leading to hybrid macrophages favorable to resolution (88).

Reparative neutrophils could also be modulated to accelerate the process of wound healing (39). In cardiac infarction, a temporal switch from inflammatory to resolving neutrophils has been detected (89). Programming neutrophils to the N2 subtype by a PPARγ agonist rosiglitazone could be used to obtain a beneficial role of anti-inflammatory N2 neutrophils, as shown in stroke (90).

### Targeting Functional Plasticity during Chronic Injury (or in Chronic Wounds)

**Figure 2:**
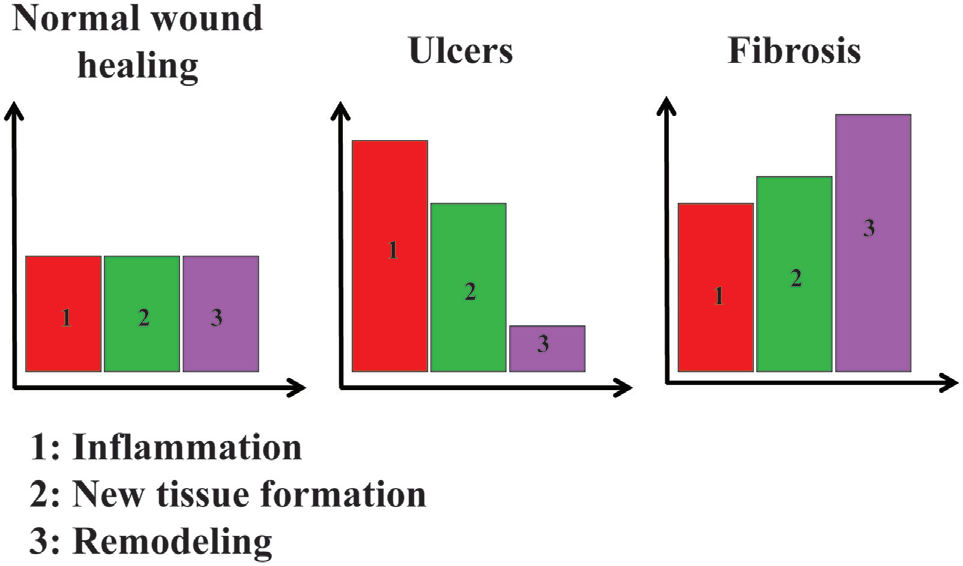
**Relative importance of inflammation, new tissue formation, and remodeling in three different situations**. In normal wound healing, inflammation (1, in red), new tissue formation (2, in green), and remodeling (3, in purple) are at a basal level. In ulcers, inflammation and new tissue formation are at higher levels, whereas remodeling is lower than the basal level. In fibrosis, inflammation, new tissue formation, and remodeling are all higher than their basal levels.

A chronic wound could be seen as resulting from a dysregulated repair process, with an increase of pro-inflammatory environment at the expense of the pro-resolving one (Figure 2). Modulating cell plasticity toward a more resolving phenotype appears an attractive strategy in that line.

Mechanical debridement of a chronic wound consists in the removal of dead tissues to improve the healing potential of the remaining healthy tissue. This removal leads to tissue re-colonization by immune cells, suggesting that they are important in the repair process. Maggot therapy could empirically modulate immune cell plasticity in addition to its mechanical role. Some data indicate that maggot secretions decrease the elastase release and the H_2_O_2_ production of activated neutrophils without affecting their phagocytic activity (91). Moreover, maggot secretions inhibit the production of pro-inflammatory cytokines by monocytes, skewing their phenotype from a pro-inflammatory to a pro-angiogenic type (92).

Another approach is to figure out the exact cause of the chronic pro-inflammatory stimulation and develop a therapeutic strategy specific to this cause. In some cases, the pro-inflammatory stimulation is associated with bacterial biofilms (93–95). Biofilms in acute surgical and chronic wounds appear to cause a delay in healing (95). In this composite state, bacteria are resistant against antibiotic treatment and immune responses, leading to impaired eradication of opportunistic pathogens. Biofilm-embedded bacteria release virulence factors dictated by quorum-sensing that in turn promote inflammatory mediators such as IL-1β, contributing to delayed wound re-epithelialization and healing (96, 97). Hence, dealing with biofilms has become a major challenge in chronic wound healing. Quorum-sensing blockers could be an innovative approach to treat non-healing wounds, even though clinical trials are needed to prove their relevance (98).

### Targeting Plasticity in Over-Repair and Fibrotic Processes

Keloid and hypertrophic scars are pathologic fibroproliferative disorders characterized by an excess of collagen deposition in genetically predisposed patients. In hypertrophic scars, inflammatory genes are expressed at a lower level and for a longer time, with a delayed but prolonged infiltration of M2 macrophages (99). Hypertrophic scar development is dependent on macrophages as their depletion during the subacute phase allows normal scarring in mice (100). Thus, limiting M2 activation in keloid could be a way to circumvent the hypertrophic scar.

Diffuse cutaneous systemic sclerosis (dcSSc) is a form of over-repair. SSc results from the interaction of three processes: innate and adaptive immune abnormalities, vasculopathy, and fibroblast dysfunction leading to excessive collagen and matrix components accumulation (101). Fibrotic skin is characterized by an immune cell infiltrate of various nature (15, 102–104). These cells follow a chemokine gradient, such as CCL2, partly explaining the recruitment of macrophages and the M2 polarization in SSc skin (105, 106). Limiting M2 activation and even activating M1 could be an interesting lead for dcSSc at the proper stage. The window of opportunity is critical, and studies showing the evolution of cell plasticity during SSc evolution are lacking to establish reliable therapy based on cell plasticity. Nevertheless one can assume that the number of pro-inflammatory innate cells is limited to a first phase, and then a pro-reparative profile of cells is predominant, giving a place for anti-resolving cell drugs. At the last stage, the absence of infiltrating cells could prevent the efficiency of immunological approaches.

## Conclusion

Immunologists have tended to see the immune system as a system of defense. Yet the immune system is crucial for several “housekeeping” processes, most prominently repair (7). In some cases, it would even seem artificial to distinguish between “repair” and “defense,” because a typical repair response (e.g., type 2) is used to “wall off” particular pathogens such as helminthes (107). By constantly surveying the body and responding to anomalies and through its pleiotropic roles in defense, development, and repair, the immune system is pivotal for the robustness of the organism (6). A system is “robust” when it is able to maintain its functions and performances despite perturbations (2–4). Robustness does not mean an absence of change: quite the contrary, it is through constant internal modifications that an organism can maintain its functions. We should therefore not be surprised by the main conclusion of the present review, which is that immune cell plasticity [and cell plasticity more generally (65, 108)] is essential to ensure the robustness of the organism as far as tissue repair is concerned.

Though still in its infancy, the idea of therapeutically manipulating immune cell plasticity in repair seems extremely promising. We have examined several examples where the manipulation of immune cell plasticity is already a reality, and we can only look forward to future investigations. Recently, an increasing number of tissue repair specialists have become interested in how non-mammal model organisms repair and/or regenerate (9). Crucially, the immune system plays a key role in repair and regeneration across species, and regeneration is often associated with an immunosuppressive state (109–111). Successful regeneration also presupposes cell plasticity, both intra-lineage and trans-lineage (112, 113). It is exciting to speculate that immune cell plasticity could play an important role in regeneration and that one day clinicians could manipulate this immune plasticity to skew the balance between damaging and reparative effects toward the desirable state for any given patient.

## Author Contributions

PL, VJ, PM, LC, CC-B, M-ET, and TP wrote the paper.

## Conflict of Interest Statement

The authors declare that the research was conducted in the absence of any commercial or financial relationships that could be construed as a potential conflict of interest.
